# The Acute Effects of Furosemide on Na-K-Cl Cotransporter-1, Fetuin-A and Pigment Epithelium-Derived Factor in the Guinea Pig Cochlea

**DOI:** 10.3389/fnmol.2022.842132

**Published:** 2022-03-22

**Authors:** Jesper Edvardsson Rasmussen, Patrik Lundström, Per Olof Eriksson, Helge Rask-Andersen, Wei Liu, Göran Laurell

**Affiliations:** Otorhinolaryngology and Head and Neck Surgery, Department of Surgical Sciences, Uppsala University, Uppsala, Sweden

**Keywords:** furosemide (frusemide), NKCC1 = Na^+^-K^+^-2Cl^—^ cotransporter, type II fibrocyte, fetuin-A, PEDF, stria vascularis, organ of corti (OoC), spiral ganglion neurons

## Abstract

**Background:**

Furosemide is a loop diuretic used to treat edema; however, it also targets the Na-K-Cl cotransporter-1 (NKCC1) in the inner ear. In very high doses, furosemide abolishes the endocochlear potential (EP). The aim of the study was to gain a deeper understanding of the temporal course of the acute effects of furosemide in the inner ear, including the protein localization of Fetuin-A and PEDF in guinea pig cochleae.

**Material and Method:**

Adult guinea pigs were given an intravenous injection of furosemide in a dose of 100 mg per kg of body weight. The cochleae were studied using immunohistochemistry in controls and at four intervals: 3 min, 30 min, 60 min and 120 min. Also, cochleae of untreated guinea pigs were tested for Fetuin-A and PEDF mRNA using RNAscope^®^ technology.

**Results:**

At 3 min, NKCC1 staining was abolished in the type II fibrocytes in the spiral ligament, followed by a recovery period of up to 120 min. In the stria vascularis, the lowest staining intensity of NKCC1 presented after 30 min. The spiral ganglion showed a stable staining intensity for the full 120 min. Fetuin-A protein and mRNA were detected in the spiral ganglion type I neurons, inner and outer hair cells, pillar cells, Deiters cells and the stria vascularis. Furosemide induced an increased staining intensity of Fetuin-A at 120 min. PEDF protein and mRNA were found in the spiral ganglia type I neurons, the stria vascularis, and in type I and type II fibrocytes of the spiral ligament. PEDF protein staining intensity was high in the pillar cells in the organ of Corti. Furosemide induced an increased staining intensity of PEDF in type I neurons and pillar cells after 120 min.

**Conclusion:**

The results indicate rapid furosemide-induced changes of NKCC1 in the type II fibrocytes. This could be part of the mechanism that causes reduction of the EP within minutes after high dose furosemide injection. Fetuin-A and PEDF are present in many cells of the cochlea and probably increase after furosemide exposure, possibly as an otoprotective response.

## Introduction

Furosemide is a loopdiuretic widely used in the treatment of edema in patients with congestive heart failure, liver failure, or kidney disease. It has an ototoxic effect if used in high doses (Schwartz et al., 1970; Santos and Nadol, 2017; Robertson et al., 2019). Loopdiuretics cause diuresis and lower blood pressure by inhibiting Na^+^-K^+^-2Cl^−^ cotransporters 1 and 2 (NKCC1, NKCC2) in the loop of Henle in the kidney, thus increasing the loss of Na^+^, K^+^, and water in the urine. NKCC1 is expressed in many tissues in the body and is also present in the inner ear (Crouch et al., 1997), while NKCC2 is kidney-specific (Delpire and Gagnon, 2018). In the inner ear, furosemide is reported to inhibit NKCC1 in the stria vascularis (Shindo et al., 1992).

Due to the greatly increased risk of synergistic damage to the cochlea, furosemide is not suitable for use with other ototoxic drugs such as aminoglycosides and cisplatin (Laurell and Engström, 1989; Alam et al., 1998; Hirose and Sato, 2011; Li et al., 2011). Furosemide reduces the endocochlear DC potential (EP) generated in the stria vascularis (Kusakari et al., 1978; Asakuma and Snow, 1980; Sewell, 1984). EP is the driving force that allows K^+^ to swiftly enter the hair cells in response to sound stimulus (Tasaki and Spyropoulos, 1959) initiating the first step of otoacoustic neurotransmission. The prolonged treatment of experimental animals with furosemide induces edema and intercellular vacuoles in the marginal cells of the stria vascularis and eventually hair cell loss in the organ of Corti (Forge, 1976; Pike and Bosher, 1980; Forge and Brown, 1982; Rarey and Ross, 1982; Naito and Watanabe, 1997). Postmortem studies have reported similar changes in the human cochlea (Arnold et al., 1981; Santos and Nadol, 2017). EP decreases in experimental animals within a few min of a high dose of furosemide, and recovers almost completely after 120 min (Kusakari et al., 1978; Asakuma and Snow, 1980; Sewell, 1984). The morphological changes in the stria vascularis induced by furosemide are observed much later. The exact mechanism behind the initial rapid EP loss is not known. Vasoconstriction and anoxia have been proposed as mechanisms involved in the initial loss of EP in experiments using another loop diuretic (Ding et al., 2002, 2016).

Fetuin-A, also known as alpha-2-HS-glycoprotein (*AHSG*), is a protein belonging to the cystatin super family synthesized in the liver and adipose tissue. Its best-known functions are the regulation of bone mineralization and protection against extra osseous calcium phosphate deposits by binding calcium phosphates (Jahnen-Dechent et al., 1997). Fetuin-A also plays a part in the anti-acute phase response (Lebreton et al., 1979; Wang and Sama, 2012), plaque formation in arteriosclerosis (Westenfeld et al., 2009; Trepanowski et al., 2015) and insulin resistance (Trepanowski et al., 2015).

Pigment epithelium-derived factor (PEDF), also known as Serpin-F1 (*SERPINF1*), is a neuroprotective, neurotrophic, and anti-angiogenetic protein first identified in the retinal pigment epithelia (Tombran-Tink et al., 1991). It is also reported to have regulating functions in osteogenesis, to promote stem cell renewal and inhibit tumor angiogenesis (Brook et al., 2020). PEDF has been identified in the stria vascularis, spiral ganglion, neurons, and basilar membrane in the rat inner ear (Gleich and Piña, 2008).

We previously reported that Fetuin-A and PEDF are part of the human perilymph (Edvardsson Rasmussen et al., 2018) and the endolymphatic sac endolymph proteome (Ölander et al., 2021). It is not known whether Fetuin-A is expressed in any cells of the inner ear or if it only appears extracellularly in the perilymph and endolymph.

The aim of the study was to gain a deeper understanding of the temporal course of the acute effects of furosemide in the inner ear, including the protein localization of Fetuin-A and PEDF in guinea pig cochleae.

## Material and Methods

### Experimental Design

Using a guinea pig animal model, protein localization and staining intensity in the cochlea was studied after an intravenous (IV) injection of 100 mg/kg body weight of furosemide. This dose is previously known to abolish the EP (Kusakari et al., 1978). The following intervals were used: 3 min, 30 min, 60 min and 120 min. Three guinea pigs were used for each interval and as controls. The guinea pigs were anesthetized, intravenously injected with furosemide and then decapitated at the desired point in time. The cochleae were quickly dissected from the temporal bone and fixated. The cochleae were cryosectioned, stained using the immunofluorescence technique and photographed with a confocal microscope. Sections from three guinea pigs without furosemide exposure were also examined using RNAscope^®^ technology. Image analysis densitometry was performed in ImageJ to semi-quantify the protein staining intensity.

### Animals

Adult (age 6–9 weeks) albino guinea pigs of both sexes (body weight 262–310 g) were used in the experiment, 15 guinea pigs for immunohistochemistry and three for RNAscope examination. The animals were housed in an enriched environment with 12/12-h day and night cycle and a temperature of 21°C and 60% humidity. They had free access to food and water. All animal procedures were performed in accordance with local ethical guidelines at Uppsala University and national legislation and regulation concerning the care and use of laboratory animals.

### Furosemide Administration

The animals were deeply anesthetized using ketamine (40 mg/kg, intramuscularly; Pfizer AB, Sweden) and xylazine (10 mg/kg, intramuscularly; Bayer, Denmark). Ophthalmic ointment was applied to the eyes to prevent corneal ulceration. The animals were given a local anesthetic by subcutaneous injection of bupivacaine hydrochloride (2.5 mg/ml) before exposure of the internal jugular vein, which was used for the IV injection. In total 12 guinea pigs were injected with 100 mg/kg of furosemide IV and were sacrificed by decapitation at four different intervals. Three control animals received the same anesthesia but were not given the furosemide injection.

### Sample Preparation

After decapitation, the temporal bone was removed and the bulla opened to expose the cochlea. Small fenestrations were performed in the apex and the round window (RW) within minutes and the cochlea was gently flushed with a 4% formaldehyde solution stabilized with phosphate buffer. The cochlea was immersed in 4% formaldehyde for 24 h and then in 0.5% formaldehyde until decalcification in 0.1 M Na-ethylenediaminetetraacetic acid (EDTA). After decalcification the cochlea was rinsed and placed in a 15% sucrose solution for 24 h followed by a gradual infiltration of 15% sucrose and Tissue-Tek Optimal Cutting Temperature (OCT) Cryomount (Histolab, Sweden) for 4 days. Finalized by infusion of pure OCT overnight, after which the cochlea was embedded in OCT. The cochlea was cryosectioned with a microtome into 8 μm thick sections throughout the cochlea and mounted on Super Frost Plus slides (Menzel-Gläser, Braunschweig, Germany), and stored in a freezer at −70°C prior to immunohistochemistry (IHC) preparation.

### Immunohistochemistry

Cochlear sections were stained according to the following protocol. Sections were rinsed three times in a glass slide staining jar with 0.01 M Phosphate Buffer Saline (PBS) with pH7.4 (Medicago) for 5 min (3 × 5 min). They were then incubated in 0.4% triton X-100 diluted in PBS at room temperature (RT) for 30 min and rinsed in PBS (3 × 5 min). The sections were incubated with primary antibodies diluted in 2% bovine serum albumin (BSA) in a humidified atmosphere at 4°C for 20 h. A negative control section was at the same time incubated with 2% BSA without primary antibody (Burry, 2011). Surplus primary antibody solution was carefully removed, and the slides were rinsed with PBS (3 × 5 min). All the sections, including negative control, were incubated with secondary antibody conjugated to Alexa Fluor 405, 488, 555, and 670 (Thermo Fisher Scientific, Sweden) for 2 h under RT. Slides were rinsed with PBS (3 × 5 min). Counterstaining was performed with the nuclear dye DAPI (4’,6-diamidino-2-phenylindole dihydrochloride) for 5–7 min at RT, after which slides were rinsed with PBS (3 × 5 min). Mounting was done with ProLong^®^ Gold or ProLong^®^ Glass Antifading Mountant and cover slipped with the specified cover glass (0.17 ± 0.005 mm) for optically matching confocal and super-resolution (SIM) microscopes. At least one representative section from each animal was selected for analysis of immunohistochemistry after all the confocal images were assessed. Images from basal or mid turn were selected since the protein localization and intensity were uniform in between the turns. Antibodies used for immunohistochemistry are listed in Table 1.

**Table 1:** Immunohistochemistry antibodies.

Antibody	Type	Species reactivity	Dilution	Host	Catalogue number	Producer
Fetuin-A	P	GP	1:100	Rabbit	ABIN2778140	Antibodies-online.com
PEDF	P	H, M	1:50	Rabbit	NBP2–19767	Novus biological
NKCC1	P	H, M, R	1:100	Rabbit	Ab59791	Abcam
Parvalbumin	M	H, M, R, P	1:200	Mouse	MAB1572	Merck
Tubulin β3	M	H, M, R, P etc.	1:200	Mouse	MAB1637	Merck

*List of antibodies used for imunohistochemistry. Type: Polyclonal (P); monoclonal (M). Species reactivty: guinea pig (GP); human (H); mouse (M); rat (R); porcine (P)*.

### RNAscope Protocol

RNA *in situ* hybridization (ISH) trials were performed using RNAscope^®^. The frozen fixed (4% paraformaldehyde) cochlear tissue sections were prepared according to the manufacturer’s instructions with the RNAscope^®^ Reagent Kit (Bio-Techne, Minneapolis, USA) (kit version 2). Sections were briefly pretreated with H_2_O_2_ (10 min, RT) and protease III (30 min, 40°C). After protease III incubation, the sections were subjected to RNAscope hybridization assay. The paired double-Z oligonucleotide probes were designed and produced by Bio-Techne based on the targets’ gene ID. To start the hybridization, the RNA probe fluid was added to the slide with sections. Incubation continued in a HybEZ^TM^ Oven (Bio-Techne) for 2 h at 40°C. After hybridization incubation, the slides were washed using 1× RNAscope^®^ Wash Buffer. Sections were then incubated with RNAscope^®^ Multiplex FL v2 Amp 1, Amp 2, and Amp 3 (for 30, 30, and 15 min respectively) sequentially at 40°C to amplify the signal. For signal development, RNAscope^®^ Multiplex FL v2 hP-C1, HRP-C2 and HRP-C3 were added to the sections sequentially (incubation time 15 min each). For detecting signals, TSA-diluted Opal^TM^ 520, 570, and 690 fluorophores were added to sections after HRP-C1, C2, and C3, incubating the sections for 30 min at 40°C for each HRP-fluorophore pare. Each of the three fluorophore incubations was followed by washing with 1× RNAscope^®^ Wash Buffer. Multiplex FL v2 hP blocker, specific for each channel, was added and incubated in the oven at 40°C for 15 min. Finally, the sections were counterstained with DAPI and the slides cover slipped with ProLong^®^ Glass Antifade Mountant (Thermo Fisher Scientific). RNAscope ISH produces puncta of signal that represent a single mRNA transcript (Grabinski et al., 2015).

A DapB probe was used for negative control. DapB is only present in a very rare strain of soil bacteria and should not produce any signal in the tissue, hence it’s utility as a negative control. Our RNAscope negative control result was consistent with the RNAscope technical protocol. The probes used for RNAscope are listed in Table 2.

**Table 2:** RNAscope probes.

Protein	Species	Gene	Gene ID	Probe	Producer
PEDF	Guinea pig	*SerpinF1*	100216362	874151	BioTechne
Fetuin-A	Guinea pig	*AHSG*	100135479	897851-C2	BioTechne

*List of probes used for RNAscope^©^*.

### Imaging and Photography

Confocal laser scanning microscopy was performed using a Nikon TE2000 inverted fluorescence microscope equipped with a three-channel laser emission system with three emission spectra filters (maxima 358, 461, and 555 nm). Confocal images were acquired using a Nikon EZ-C1 (ver. 3.80) software, with all acquisition settings kept equal within each figure, except for the blue (DAPI) channel which was optimized for illustration of the morphology. The Nikon EZ-C1 was also used for reconstructions of z-stacks to 3D-images. The confocal images were saved as tag image file format red-green-blue (TIFF-RGB) with a resolution of 512 × 512 pixels and transferred to Fiji ImageJ 1.53C (Schindelin et al., 2012). Fiji ImageJ was used to perform densitometry. The image was split into channels for red, green, and blue immunostaining. The channel of interest was isolated and converted into a grayscale image in which each pixel was assigned a value between 0 and 65,535 depending on the intensity of immunofluorescence, where 0 is black and 65,535 is white. The grayscale image was used for intensity measurement. A region of interest was manually drawn to exclude areas of the sample that were not subject to examination. The threshold for minimum intensity required of a pixel to be included was set according to “Li” to remove background pixels (Li and Lee, 1993; Li and Tam, 1998). Fiji ImageJ measured the histogram of the remaining pixels and their intensity value. The mean intensity value of the remaining pixels was calculated. All immunohistochemistry images selected for analysis in controls and at 120 min were included in densitometry analysis. Densitometry values were calculated for one representative confocal image from each animal in the controls and 120 min group, and presented as mean values with error bars for standard deviation. Counting and rating of the nuclei staining of Fetuin-A was done in one representative confocal image from each animal in the five groups. The staining of the nuclei of the marginal cells of the stria vascularis and the type I neurons of the spiral ganglia were rated as no, weak or strong intensity. Mean and standard deviation was calculated.

## Results

### Longitudinal Pattern of Immunostaining

Patterns of immunohistochemistry were studied at the four intervals and compared to control. Densitometry was calculated for 0 and 120 min for Fetuin-A and PEDF immunohistochemistry and Fetuin-A nuclei staining was rated and counted. The presence of Fetuin-A mRNA and PEDF mRNA in guinea pigs not exposed to furosemide were analyzed using RNAscope. The protein localization and signal intensity as well as mRNA detection were uniform in the different turns of the cochlea when the sections were studied visually. The results are presented below for the different compartments of the cochlea.

### The Lateral Cochlear Wall

**Figure 1:**
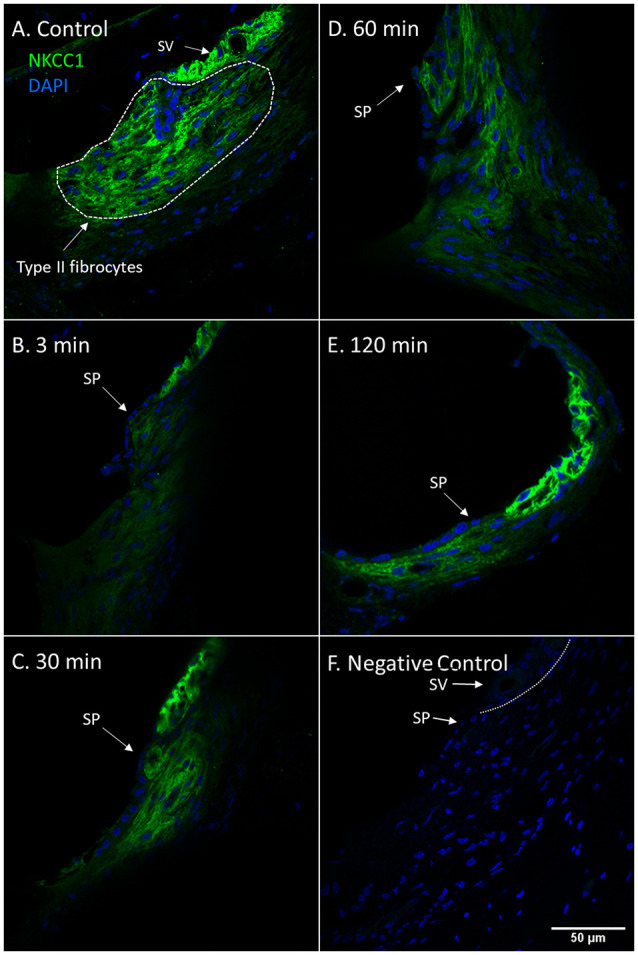
NKCC1 in type II fibrocytes following furosemide injection. **(A–F)** Staining intensity of NKCC1 in the type II fibrocyte following administration of furosemide with DAPI staining of cell nuclei. Focus was set on the type II fibrocytes. Spiral prominence (SP); Stria vascularis (SV). **(A)** Normal NKCC1 staining in type II fibrocytes in a control guinea pig. **(B)** At 3 min after injection there was almost no staining of NKCC1 in the type II fibrocytes. **(C)** At 30 min the staining intensity of NKCC1 in the type II fibrocytes had returned to a low level. **(D)** At 60 min staining intensity was still decreased compared to control. **(E)** After 120 min the signal intensity had returned to near initial levels. **(F)** Negative control section with DAPI.

NKCC1 is known to be localized to the baso-lateral wall of the marginal cells in the stria vascularis (Crouch et al., 1997) and the type II fibrocytes of the spiral ligament (Spicer and Schulte, 1991). The control animals showed strong NKCC1 staining intensity in the marginal cells of the stria vascularis and type II fibrocytes in the spiral prominence region. Confocal microscopy revealed a drastic reduction of NKCC1 staining intensity in the type II fibrocytes at 3 min compared to the control. A progressive increase of NKCC1 staining intensity followed at the subsequent intervals in the type II fibrocytes until 120 min when NKCC1 staining intensity had nearly recovered to control levels (Figure 1).

**Figure 2:**
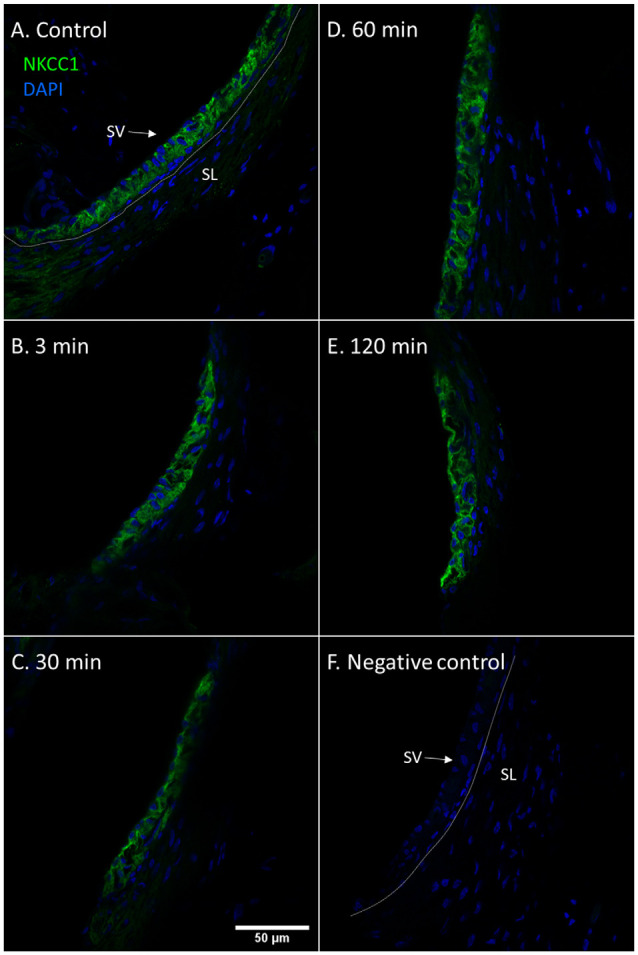
NKCC1 in the stria vascularis following furosemide injection. **(A–F)** Staining intensity of NKCC1 in the marginal cells of the stria vascularis following furosemide injection with DAPI staining of nuclei. Focus was set on the stria vascularis. Spiral ligament (SL); Stria vascularis (SV). **(A)** Normal NKCC1 staining in the stria vascularis in a control animal. **(B)** Intensity had decreased after 3 min. **(C)** The lowest intensity was seen at 30 min. **(D)** It then recovers partially at 60 min. **(E)** Further recovery at 120 min. **(F)** Negative control with DAPI.

The marginal cells of the stria vascularis had stronger NKCC1 staining intensity in the controls and at all intervals following furosemide injection compared to the type II fibrocytes. The staining intensity of NKCC1in the stria vascularis was decreased at 3 min, but was prominently lower at 30 min. Thereafter the staining intensity of NKCC1 gradually recovered in the stria vascularis (Figure 2).

**Figure 3:**
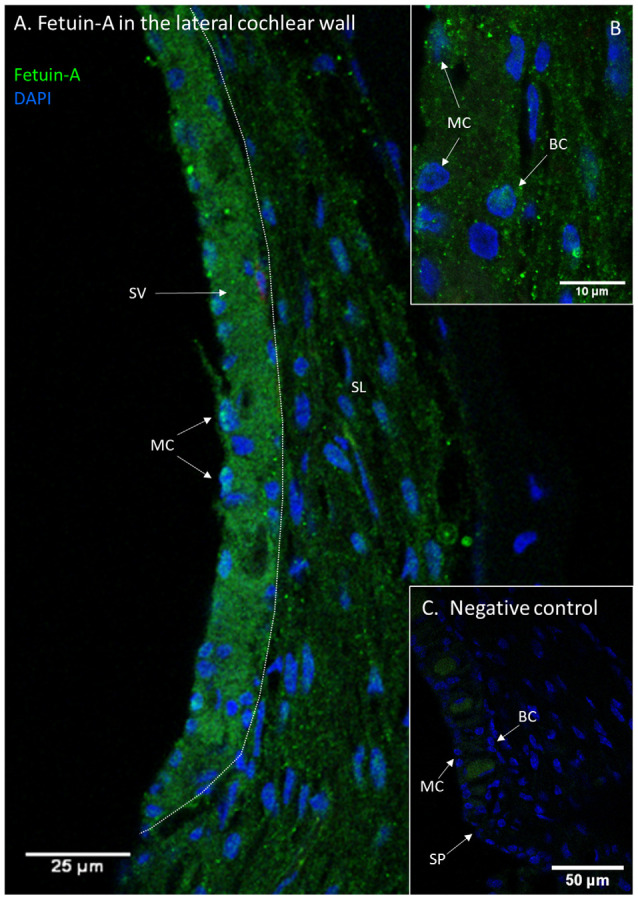
Fetuin-A in the lateral cochlear wall. Protein localization of Fetuin-A in the lateral cochlear wall of a control guinea pig. Spiral ligament (SL); Stria vascularis (SV); Spiral prominence (SP); Marginal cell (MC); Basal cell (BC). **(A)** Fetuin-A protein in the spiral ligament, spiral prominence, and stria vascularis. **(B)** Inset of higher magnification of stria vascularis. Marginal cells (MC) and also basal cells (BC) have Fetuin-A protein. **(C)** Negative control of lateral wall with stria vascularis, spiral prominence and spiral ligament. Vessels have a weak unspecific staining.

**Figure 4:**
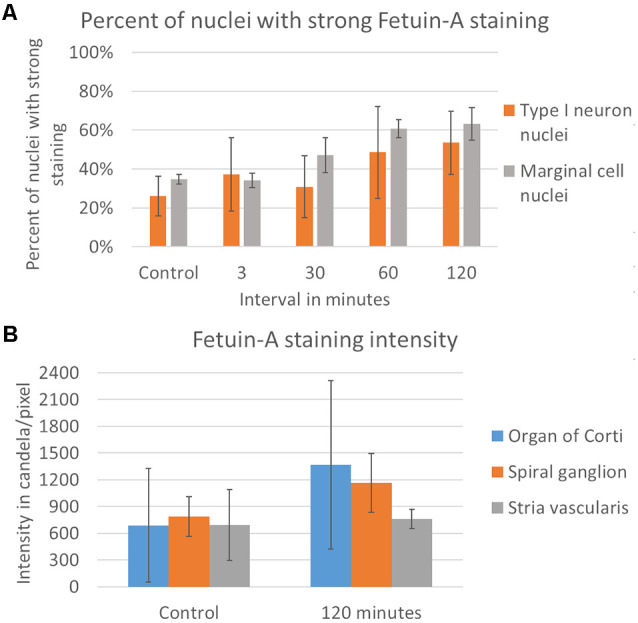
Rating of Fetuin-A nuclei staining and densitometry. **(A)** Rating of Fetuin-A staining intensity in type I neuron nuclei (orange) and marginal cell nuclei (gray). Result presented as the mean value of each group, with error bars showing standard deviation. **(B)** Densitometry of Fetuin-A staining intensity. Result presented as mean value of the control and 120 min group, with error bars showing standard deviation. Intensity increased in the organ of Corti (blue) and the spiral ganglion (orange), with no change in the stria vascularis (gray).

Fetuin-A protein was detected with a low and consistent staining intensity in the stria vascularis, spiral ligament and bone surrounding the cochlear structures in the controls. The stria vascularis had the strongest staining intensity of the different compartments in the lateral wall, and some of the marginal cells’ nuclei showed Fetuin-A staining (Figure 3). The percentage of marginal cell nuclei with strong Fetuin-A staining increased between 30 min and 120 min after furosemide injection. In the control group had 35% of the marginal cells’ nuclei a strong staining intensity, which was in the 120 min group increased to 63%. However, the mean staining intensity of stria vascularis did not change (Figure 4).

**Figure 5:**
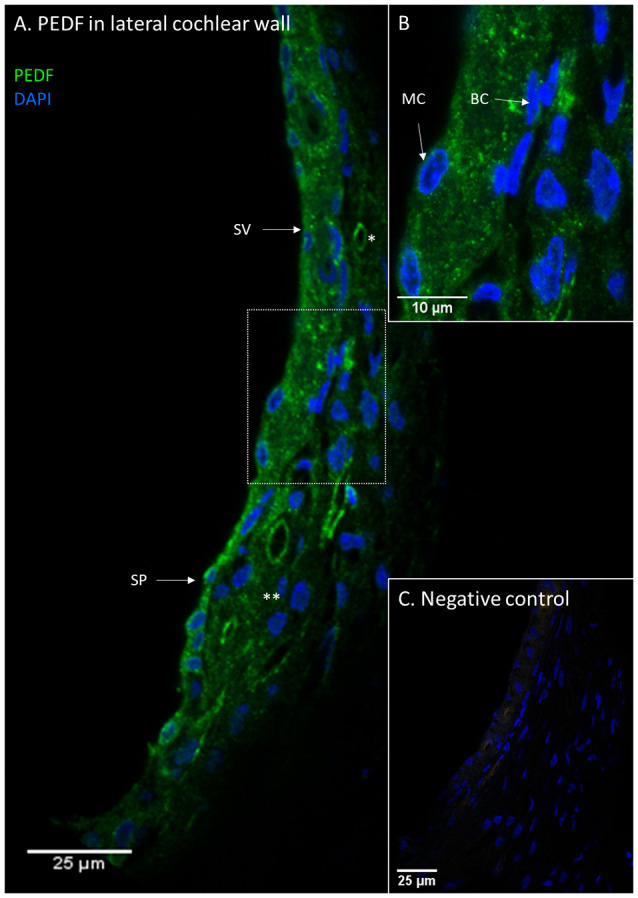
PEDF in the lateral cochlear wall. PEDF protein localization in the lateral cochlear wall. Stria vascularis (SV); Spiral prominence (SP); Marginal cell (MC); Basal cell (BC); type I fibrocytes (*); type II fibrocytes (**). **(A)** PEDF was localized to the spiral ligament, the stria vascularis and the spiral prominence. In the spiral ligament were type I and II fibrocytes PEDF positive. **(B)** Inset of higher magnification of marked area in **(A)**. The distribution of PEDF in sub-cellular level of stria vascularis and spiral ligament. **(C)** Negative control of the immunohistochemistry.

**Figure 6:**
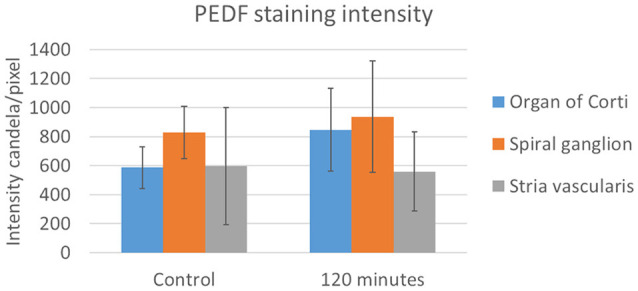
PEDF in the stria vascularis following furosemide injection. Densitometry of PEDFstaining intensity. Result presented as mean values of the control and 120 min group, with error bars showing standard deviation. The organ of Corti (blue) showed a large increase and in the spiral ganglion (orange) a marginal increase, while the stria vascularis (gray) had no change.

PEDF protein was detected in the stria vascularis, spiral prominence epithelia and type I and type II fibrocytes, while the type III, IV, and V fibrocytes were negative. The stria vascularis was positive for PEDF in all cell layers. PEDF staining intensity was similar in the spiral prominence and in the stria vascularis (Figure 5). There were no visual differences in PEDF protein localization or staining intensity in the lateral wall after furosemide exposure compared to the controls (Figure 6).

### The Spiral Ganglion

NKCC1 was detected in the cell membrane of the spiral ganglion neurons in all controls and furosemide exposed animals, with no difference during the studied period of 120 min.

**Figure 7:**
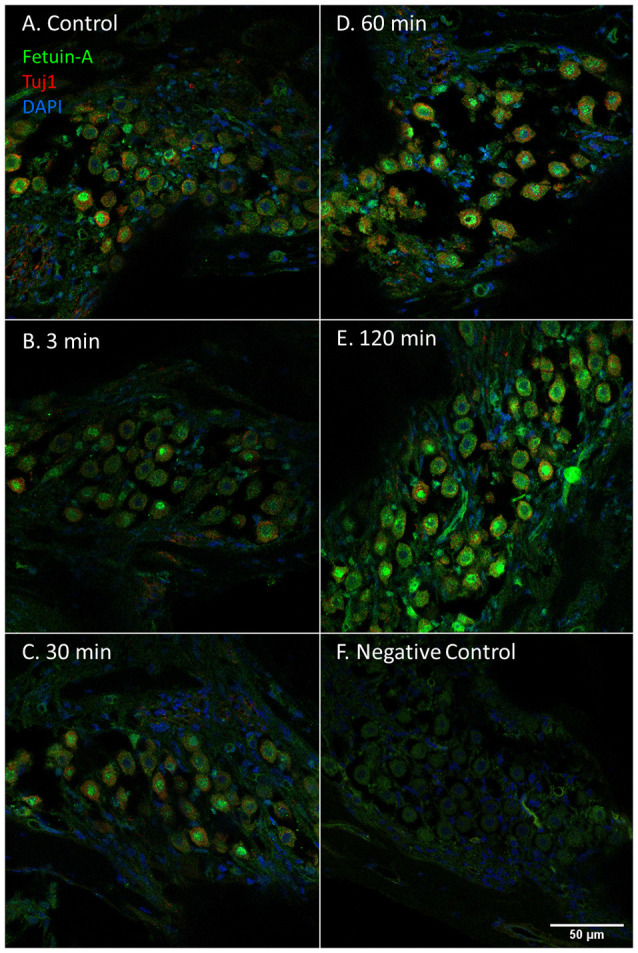
Fetuin-A in the spiral ganglion following furosemide injection. **(A–F)** Fetuin-A protein staining of guinea pig spiral ganglion following IV furosemide injection. **(A)** Fetuin-A protein was localized to the type I neurons cytoplasm and nuclei in the control. **(B–E)** The staining intensity after furosemide at different intervals. **(F)** Negative control.

Fetuin-A protein was seen in the cytoplasm and nuclei of the spiral ganglion type I neurons (Figure 7). The mean percent of type I neuron cell nuclei with strong Fetuin-A staining was 26% in the controls. This was increased to 49% at 60 min, and 53% at 120 min (Figure 4). The mean intensity of the total staining in the spiral ganglion measured with densitometry in controls and at 120 min was presented in Figure 6.

**Figure 8:**
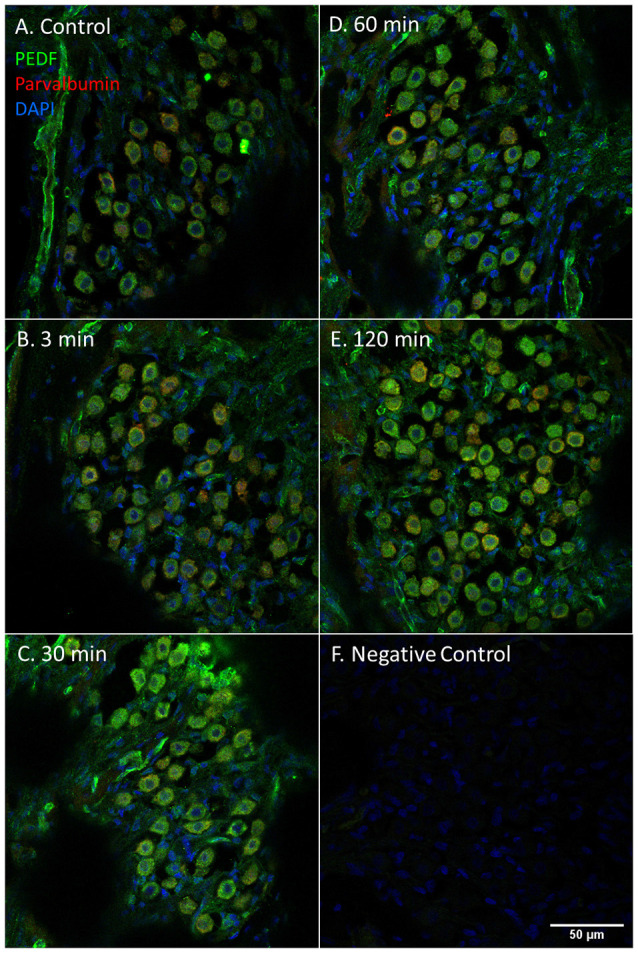
PEDF in the spiral ganglion following furosemide injection. **(A–F)** PEDF protein staining of guinea pig spiral ganglion following IV furosemide injection. **(A)** In the control was PEDF localized in the cytoplasm of the spiral ganglion type I neurons. **(B–E)** The staining intensity after furosemide at the different intervals. **(F)** Negative control.

PEDF protein was detected in the cytoplasm of the type I neurons in the spiral ganglion in the control and at all intervals after furosemide administration (Figure 8). The signal was stable over time and densitometry indicates a minor increase in the type I neurons at 120 min (Figure 6).

### The Organ of Corti

**Figure 9:**
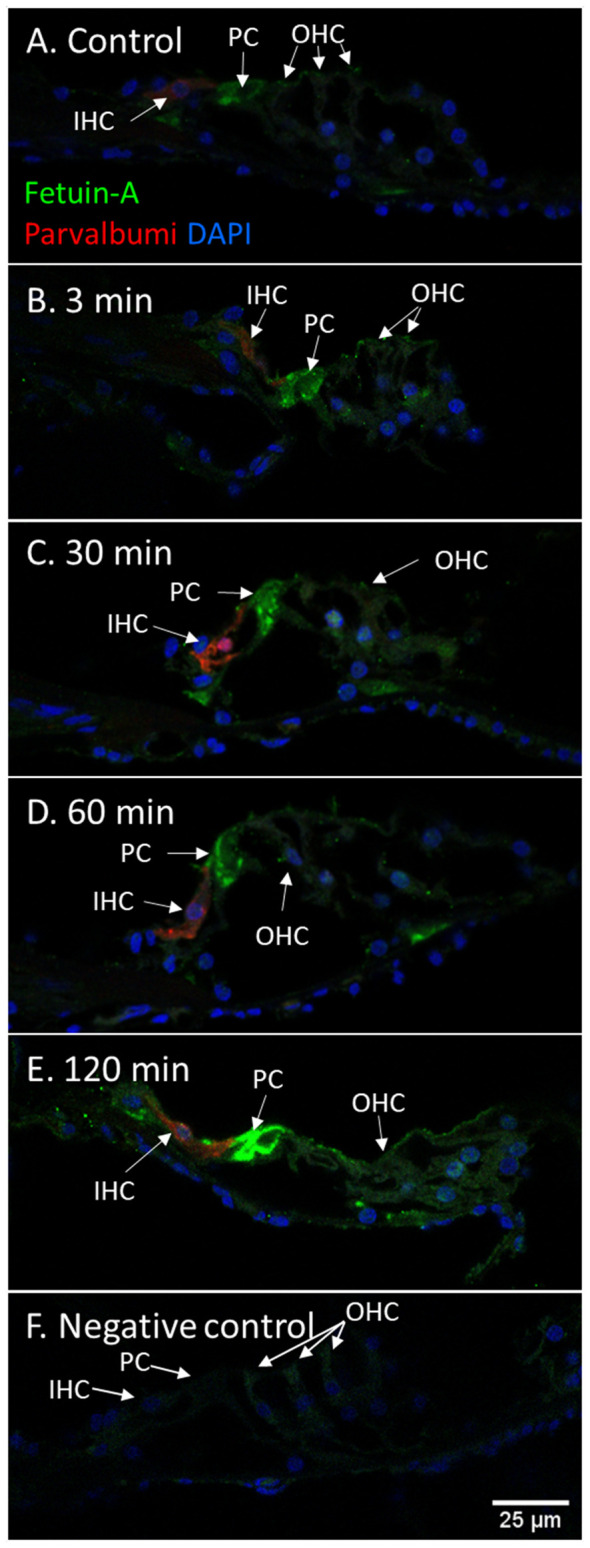
Fetuin-A in the organ of Corti following furosemide injection. **(A–F)** Fetuin-A protein localization and staining intensity in the guinea pig’s organ of Corti following IV furosemide injection. Parvalbumin stains the IHC red. **(A)** In the control was Fetuin-A detected in the pillar cells. **(B–E)** Staining intensity in the guinea pig’s organ of Corti following IV furosemide injection. Fetuin-A staining was greatly increased in the pillar cells, and the IHCs, OHCs, and Deiters were positive after 120 min. **(F)** Negative control of organ of Corti.

Fetuin-A protein was detected with a strong staining intensity in the pillar cells of the control animals. The inner and outer hair cells (IHCs and OHCs) and Deiters cells had a low staining in the control and was judged negative. However, after furosemide exposure, the signal intensity increased markedly in the pillar cells, and after 120 min also in IHCs, OHCs, and Deiters cells (Figure 9). Densitometry measurement showed an increased mean value of the signal intensity in the organ of Corti at 120 min (Figure 4).

**Figure 10:**
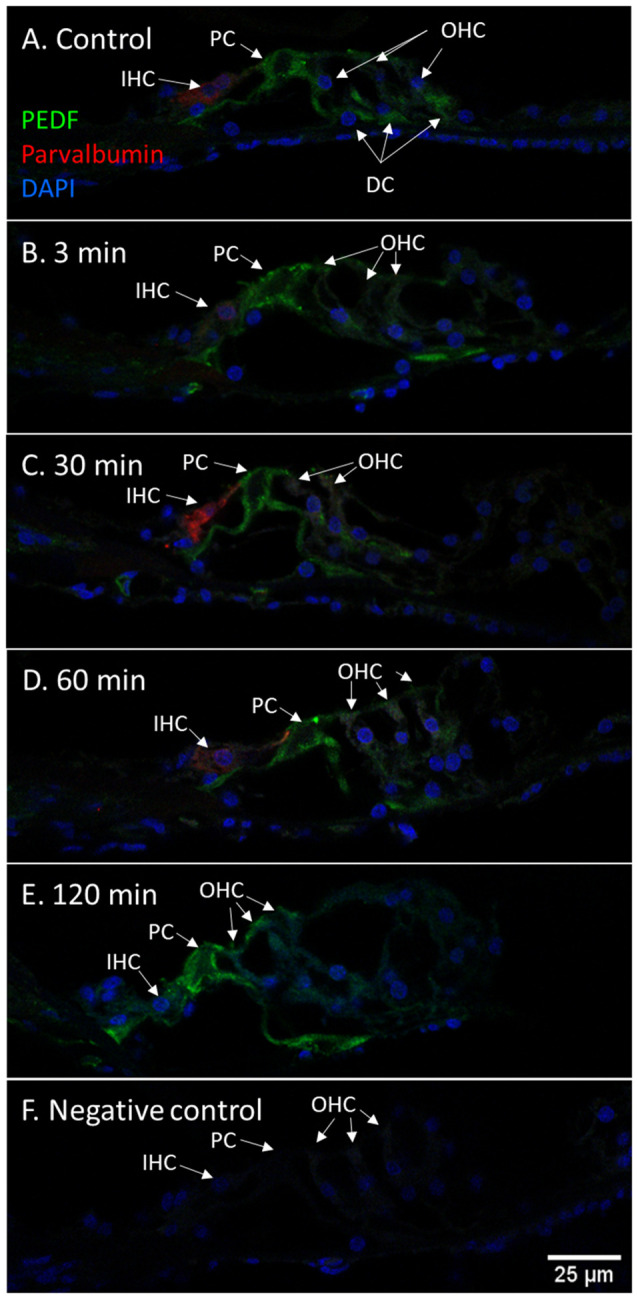
PEDF in guinea pig organ of Corti following furosemide injection. **(A–F)** PEDF protein localization and staining intensity in the guinea pig’s organ of Corti following IV furosemide injection. Parvalbumin stains the IHC red. **(A)** PEDF was in the control detected in pillar cells and Deiters cells. **(B–E)** Staining intensity in the guinea pig’s organ of Corti following IV furosemide injection. **(E)** At 120 min staining increased in the pillar cells and the IHCs and OHCs stained for PEDF protein. **(F)** Negative control of organ of Corti.

Immunohistochemistry detected PEDF protein in the pillar cells and Deiters cells in the controls. After 120 min PEDF protein were also detected in the IHCs, OHCs, and Deiter cells in the organ of Corti. The staining intensity increased most in the pillar cells. No immunostaining was observed in the basilar membrane in the guinea pig’s organ of Corti (Figure 10). Densitometry showed that the mean staining intensity of PEDF in organ of Corti had increased by 120 min and the highest staining intensity in the organ of Corti was observed in the pillar cells (Figure 6).

### RNAscope

The RNAscope technique was used in untreated guinea pigs with consistent results compared to the protein localization detected with immunohistochemistry in all cochlear compartments, except for PEDF in the organ of Corti.

**Figure 11:**
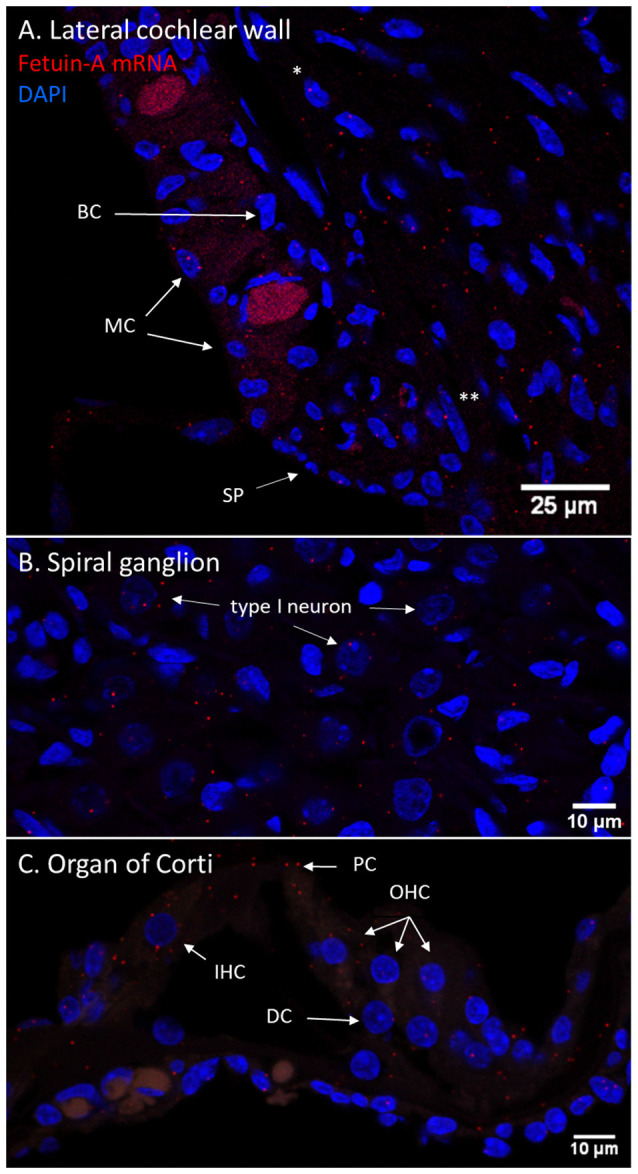
Fetuin-A m-RNA in the guinea pig cochlea. **(A–C)** RNAscope of Fetuin-A mRNA in the control guinea pig cochlea. Red dots indicate mRNA transcripts. DAPI staining of nuclei. Spiral prominence (SP); Marginal cell (MC); Basal cell (BC); type I fibrocytes (*); type II fibrocytes (**). **(A)** Fetuin-A mRNA was detected in all cell layers of stria vascularis, spiral ligament, and prominence. **(B)** The spiral ganglion type I neurons had Fetuin-A mRNA transcripts, but in small numbers. **(C)** In the guinea pig organ of Corti was Fetuin-A mRNA transcripts detected in the IHCs, OHCs, Deiters cells (DC), and pillar cells (PC). PEDF mRNA in the guinea pig cochlea.

In the cochlear lateral wall, Fetuin-A mRNA transcripts were detected in the whole stria vascularis and spiral ligament. In the spiral ganglion, Fetuin-A transcripts were detected in the type I neurons. In the organ of Corti Fetuin-A mRNA transcripts were detected in the IHCs, OHCs, pillar cells, and Deiters cells as well as Hensen’s cells and Boetcher’s cells in accordance with the findings in the furosemide groups (Figure 11).

**Figure 12:**
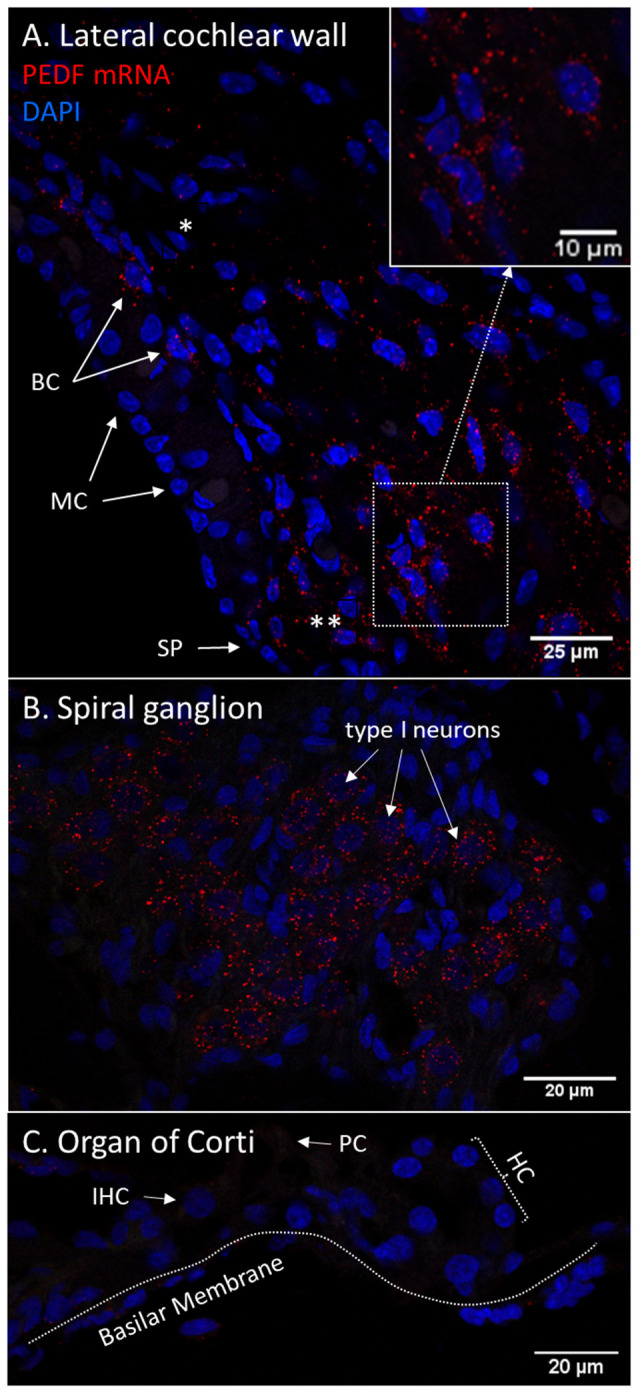
**(A–C)** RNAscope of PEDF mRNA in the normal guinea pig cochlea. Red dots indicate mRNA transcripts. DAPI staining of nuclei. Spiral prominence (SP); Marginal cell (MC); Basal cell (BC); Hensen’s cells (HC); type I fibrocytes (*); type II fibrocytes (**).** (A)** PEDF mRNA transcripts were found in the stria vascularis, spiral ligament, and spiral prominence. The type II fibrocytes had the most abundant transcripts. Transcripts were detected in type I fibrocytes in thebasal layer of the stria vascularis. **(B)** The spiral ganglion type I neurons demonstrate high numbers of PEDF mRNA transcripts. **(C)** No PEDF mRNA were found in the organ of Corti.

PEDF mRNA transcripts were found in the basal cells of the stria vascularis and in type I and type II fibrocytes. In the lateral wall, transcripts were most abundant in type II fibrocytes. In the spiral ganglion, type I neurons had abundant transcripts. No PEDF mRNA was found in the organ of Corti in the control guinea pig, despite positive protein immunohistochemistry (Figure 12).

## Discussion

### Furosemide-Induced Changes to NKCC1 Staining

This explorative study has identified a new important target and possible mechanisms of furosemide ototoxicity. Many previous experimental studies have shown that a high dose of furosemide, either alone or in combination with other ototoxic drugs, causes a rapid reduction in the endocochlear potential (EP) (Kusakari et al., 1978; Asakuma and Snow, 1980; Sewell, 1984). In the guinea pig cochleae, we observed that furosemide targets NKCC1 in type II fibrocytes in the spiral ligament almost immediately after an IV administration. The spiral ligament fibrocytes are classified into types I to V (Spicer and Schulte, 1991). The classification is based on the expression of specific ion pumps or channels and the location in the spiral ligament. The function of the spiral ligament fibrocytes is believed to be active recirculation of K^+^ from the hair cells and perilymph back to the stria vascularis (Spicer and Schulte, 1996). Disruption of the spiral ligament is known to decrease the EP and increase the threshold of the auditory brainstem response (ABR) in experimental animals (Kikuchi et al., 2000; Marcus et al., 2002; Takiguchi et al., 2013; Yoshida et al., 2015; Kitao et al., 2016). The major transporting mechanisms of K^+^ are thought to be through gap junctions and connexin channels, inward rectifying potassium channel Kir4.1 and the ion pumps NKCC1 and Na-K-ATPase (Spicer and Schulte, 1991, 1996; Weber et al., 2001; Liu et al., 2017). The type II fibrocytes are recognized by expression of Na-K-ATPase and NKCC1 (Spicer and Schulte, 1991; Crouch et al., 1997) and they are located in the spiral prominence area and outer sulcus inferior to stria vascularis. Observations in a mouse model after acute cochlear energy trauma revealed that hearing recovery was linked to re-expression of Na-K-ATPase and connexin26 (Kitao et al., 2016). Furthermore, reductions in NKCC1 and NK-K-ATPase in type II fibrocytes in addition to age-related hearing loss have been reported in Sprague-Dawley rats (Takiguchi et al., 2013). Another experiment, in which the perilymph compartment was perfused with the loop diuretic bumetanide, showed a decrease in EP at the same time as an increase in the intrastrial space, K^+^, indicating a blockade of NKCC1 in the lateral wall and marginal cells (Yoshida et al., 2015). This is in line with the present finding of immediate reduction in NKCC1 staining in type II fibrocytes, and this loss of function may be the first target of the loop diuretic effect on EP.

Previous research has shown that NKCC1 is located in the basolateral surface of marginal cells in the stria vascularis (Shindo et al., 1992; Crouch et al., 1997) and in type II fibrocyte (Spicer and Schulte, 1991). While NKCC1 staining intensity reached the lowest point in type II fibrocytes at 3 min after furosemide administration, the stria vascularis was most inhibited at 30 min after furosemide administration. This time difference might be explained by the vascular structure of the lateral wall of the cochlea. The concept of a barrier system between the blood compartment and inner ear structures is widely accepted. In analyzing the barrier systems between the blood compartment and inner ear structures, it is anatomically and functionally relevant to separate the blood-labyrinth barrier into the blood-perilymph barrier and the intrastrial fluid-blood barrier (Juhn and Rybak, 1981; Cohen-Salmon et al., 2007). The intrastrial fluid-barrier in the stria vascularis is formed by endothelial cells connected to each other by tight junctions and surrounded by a basal membrane. The next layer is formed by pericytes and perivascular resident macrophage-like melanocytes (PVM/M) around the capillaries (Juhn and Rybak, 1981; Cohen-Salmon et al., 2007; Liu et al., 2016; Shi, 2016). This barrier protects the stria vascularis and the endolymphatic compartment from exogenous substances in the circulatory system. The other parts of the spiral ligament, including the type II fibrocytes, are protected by the more permeable blood-perilymph barrier. This barrier consists of endothelial cells connected to each other by tight junctions but with a few fenestrations (Jahnke, 1980). It is known that the blood-perilymph barrier is more permeable than the intrastrial fluid-blood barrier (Sterkers et al., 1987; Juhn et al., 2001; Counter et al., 2017). Experiments on the permeability of the blood-perilymph barrier in the chinchilla demonstrate that EP loss after furosemide administration is correlated to the concentration of the drug in perilymph (Juhn and Rybak, 1981). More recent findings in an MRI study showed a much slower opening of the intrastrial fluid-barrier after the administration of a high dose of furosemide (Videhult Pierre et al., 2020) than the expected loss of EP. We, therefore, consider the observation of a more rapid loss of NKCC1 in type II fibrocytes than in marginal cells, to be consistent with previous experiments on inner ear barriers and the differences in the permeability of the barriers might be a mechanism behind the more rapid effect on the type II fibrocytes of furosemide.

The changes seen in NKCC1 staining intensity of the type II fibrocytes were very rapid and might therefore not be related to change in the gene expression (Ding et al., 2014). Regulation of NKCC1 activity has been studied in brain tissue and posttranslational mechanisms are reported to contribute to homeostatic regulation (Watanabe and Fukuda, 2020). Examples of posttranslational mechanisms contributing to the regulation of NKCC1 are glycosylation and phosphorylation. Glycosylation of the N-terminal increases membrane trafficking (Singh et al., 2015) and ion transportation (Ye et al., 2012) of NKCC1. Phosphorylation of NKCC1 by Serine/threonine-protein kinase WNK3 activates NKCC1 ion transportation and de-phosphorylationby protein phosphatase 1 inhibits ion transportation (Kahle et al., 2005). If these mechanisms can also be influenced by a high dose of furosemide has not been reported earlier. Studies on the structural molecular interaction of furosemide and NKCC1 are few. Furosemide has been reported to bind transiently to the Cl^−^ transporting site of NKCC1 (Somasekharan et al., 2012). If the binding of furosemide to the NKCC1 protein interferes with the binding of the immunohistochemistry antibody against NKCC1 or if posttranslational modifications of NKCC1 could explain the loss of signal intensity has yet to be elucidated.

### Longitudinal Fetuin-A Response

The vulnerability of the cochlea to insults induced by various ototoxic drugs has been known for decades. The combination of an aminoglycoside antibiotic and furosemide has been shown to induce an inflammatory response in the cochlea (García-Alcántara et al., 2018; Kaur et al., 2018). Whether an immune response in the cochlea can be activated by a single high dose of furosemide as used in the present study remains open to question. There has been no previous mapping of Fetuin-A in either human cochlear structures or in experimental research. In the cochleae of our control guinea pigs, Fetuin-A was present in the neurons of the spiral ganglion, pillar cells of the organ of Corti and marginal cells of the stria vascularis. Following furosemide administration, a clear temporal staining pattern for Fetuin-A was observed in the cochlea, with an increase of the protein staining intensity in all the Fetuin-A-positive cells after 60–120 min. After 120 min Fetuin-A protein was also detected in IHCs, OHCs, and Deiters cells. To further explore the Fetuin-A expression, we analyzed control guinea pig cochleae for Fetuin-A mRNA using the RNAscope method. The presence of Fetuin-A mRNA in the cells of the cochleae where Fetuin-A protein was detected, verified the results obtained by immunohistochemistry, and indicates that the Fetuin-A gene was expressed. Presence of Fetuin mRNA suggest that the observation of a time-dependent increase of Fetuin-A staining intensity in these cochlear structures may be a manifestation of local cellular activation due to furosemide ototoxicity, in contrast to an increased uptake from the systemic circulation.

Fetuin-A is a multifunctional protein known to be primarily secreted from the liver and reported to be mainly involved in anti-inflammatory mechanisms (Lebreton et al., 1979; Wang and Sama, 2012). In the early phase of an acute inflammation, Fetuin-A concentration in plasma is lowered by pro-inflammatory cytokines and increased by the late inflammation response (Wang and Sama, 2012). Fetuin-A does, however, protect against peripheral artery disease in patients with chronic kidney disease (Westenfeld et al., 2009; Jirak et al., 2019). On the other hand, Fetuin-A is shown to increase insulin resistance and arteriosclerosis in patients with metabolic syndrome (Trepanowski et al., 2015). Fetuin-A function can, therefore, be considered to be dualistic. In our earlier assessment of perilymph proteome in patients with vestibular schwannoma, Fetuin-A was found to be positively correlated with preserved hearing (Edvardsson Rasmussen et al., 2018). We therefore interpret the finding of increased Fetuin-A after injection with furosemide as a possible otoprotective function after acute insults to the cochlea.

### Longitudinal PEDF Response

PEDF protein was detected with immunohistochemistry in the lateral cochlear wall, spiral ganglion and organ of Corti in control guinea pigs. The protein was most evident in type II fibrocytes, the basal and marginal cells of the stria vascularis, the type I neurons of the spiral ganglion and in the pillar cells in the organ of Corti. After 120 min of IV furosemide administration, PEDF had increased in the type I neurons and in the pillar cells. IHCs, OHCs, and Deiters cells were also PEDF-positive after 120 min. PEDF was not detected in the basilar membrane in guinea pigs, which has been reported in rats (Gleich and Piña, 2008). PEDF gene expression was further analyzed using RNAscope in the cochleae of normal guinea pigs and we found mRNA transcripts in spiral ganglion type 1 neurons, type I and type II fibrocytes, marginal cells, and basal cells of the stria vascularis. The finding of mRNA transcripts was a first validation of the detected protein localization and indicate that PEDF gene expression was active in these cells. We could not confirm PEDF mRNA in the organ of Corti, although the immunohistochemistry protein localization was consistent in the experiment. Based on these results, we speculate that PEDF could be produced by cells in the lateral cochlear wall and can reach the organ of Corti from the endolymph. An alternative possible explanation could be that PEDF was transported by axonal transportation from the type I neurons to the organ of Corti. However, it cannot fully be ruled out that PEDF gene transcription could be very low in the organ of Corti in the normal situation and could be increased from cellular stress induced by furosemide.

PEDF has been reported in the perivascular melanocyte-like macrophages (PVM/M) (Zhang et al., 2012), part of the second layer in the intrastrial fluid-blood barrier. Further, the PEDF signaling pathway was reported to be important for the formation of tight junctions between endothelial cells to maintain the integrity of the intrastrial fluid-blood barrier after acoustic trauma (Zhang et al., 2013). The timing of the increase of PEDF staining intensity in our experiment was similar to that observed after acoustic trauma by Zhang et al. (2013). Stimulation of PEDF production was reported to increase the survival of PVM/M in cell culture and stimulate the formation of tight junctions (Zhang et al., 2021). It could therefore be part of a counter mechanism to the increased permeability of the intrastrial fluid-blood barrier caused by furosemide (Videhult Pierre et al., 2020).

Increased staining intensity of PEDF protein were observed in the organ of Corti in the pillar cells, IHCs, OHCs, and Deiters cells after 120 min. PEDF is known to promote the survival of photoreceptors and retinal ganglion cells (Barnstable and Tombran-Tink, 2004; Polato and Becerra, 2016; Zwanzig et al., 2021) and the suppression of pro-inflammatory cytokines (Ma et al., 2021). We, therefore, speculate that increased PEDF in the organ of Corti also promotes cell survival after the cochlear insult induced by furosemide.

We detected PEDF in the cytoplasm of the spiral gangliontype I neurons, and an increase in PEDF over time reaching a maximum at 60–120 min. The PEDF increase was similar to the known neuroprotective response in retinal ganglion cells (Unterlauft et al., 2012) and probably a mechanism to protect type I neurons from undergoing apoptosis.

The finding of increased Fetuin-A and PEDF in the cochlea after furosemide exposure and the information of their anti-inflammatory and protective functions suggest a possible causal effect behind the observed correlation of high Fetuin-A in perilymph and preserved hearing in vestibular schwannoma patients (Edvardsson Rasmussen et al., 2018). Given the reported links between PEDF-gene mutations and hereditary otosclerosis (Ziff et al., 2016), it is relevant to further explore PEDF in the inner ear.

It may be argued that it is difficult to draw conclusions from a longitudinal study using immunohistochemistry, as it is not possible to follow immunohistochemically changes in the inner ear over time in the same experimental animal. We, therefore, created a timeline comparing different individuals with carefully minimized experimental differences. This immunohistochemistry study was designed to gain more knowledge of time-dependent changes induced by furosemide in the cochlea. However, the number of animals used in the study did not allow for statistical analysis of the signal intensity changes. One further limitation of the study was that no *in vivo* measurements of ABRs or EP were taken.

## Conclusion

NKCC1 staining was found to be greatly reduced in type II fibrocytes of the spiral ligament 3 min after the administration of a high dose of furosemide. The NKCC1 signal followed the temporal course previously reported for EP recovery. This suggests that one underlying mechanism of EP decrease following furosemide administration could be the disruption of K^+^ recirculation *via* the type II fibrocytes into the syncytium of the stria vascularis.

PEDF and Fetuin-A protein were both present in the stria vascularis, spiral ganglion and organ of Corti in controls. The cells with high staining of both proteins were marginal cells of the stria vascularis, type I neurons and pillar cells. The presence of Fetuin-A mRNA and PEDF mRNA shows a local gene expression in the cochlea. There was a temporal pattern with increase in both Fetuin-A and PEDF 120 min after furosemide administration, indicating that these proteins may play a role in the cellular response to cochlear insults.

## Data Availability Statement

The raw data supporting the conclusions of this article will be made available by the authors, without undue reservation.

## Ethics Statement

The animal study was reviewed and approved by Ethics Committee on Animal Experiments in Uppsala, Sweden (ref. no. 5.8.18-13236/2018).

## Author Contributions

GL, PE, and JE conceived the study design and primary hypothesis. WL and JE performed the immunohistochemical staining and microscopy. WL also performed the RNAscope and microscopy. PL was responsible for the densitometry analysis. HR-A and JE interpreted the morphological results. All authors discussed the results and thereafter JE wrote the manuscript. All authors contributed to the article and approved the submitted version.

## Conflict of Interest

The authors declare that the research was conducted in the absence of any commercial or financial relationships that could be construed as a potential conflict of interest.

## Publisher’s Note

All claims expressed in this article are solely those of the authors and do not necessarily represent those of their affiliated organizations, or those of the publisher, the editors and the reviewers. Any product that may be evaluated in this article, or claim that may be made by its manufacturer, is not guaranteed or endorsed by the publisher.
